# Multimodal PSOCT-NIRS catheter for guided ablation of atrial fibrillation

**DOI:** 10.1117/1.JBO.31.4.046004

**Published:** 2026-04-06

**Authors:** Michael Douglass, Reza Mohammadpour, Walter Hoyt, Ohad Ziv, Kenneth R. Laurita, Christine Hendon, Andrew M. Rollins

**Affiliations:** a Case Western Reserve University, Biomedical Engineering Department, Cleveland, Ohio, United States; b Ochsner Health, Pediatric Cardiology, New Orleans, Louisiana, United States; c Case Western Reserve University, Heart and Vascular Research Center, MetroHealth Campus, Cleveland, Ohio, United States; d Columbia University, Electrical Engineering, New York, New York, United States

**Keywords:** ablation, probe, multimodal, cardiac, catheter, polarization, diffuse reflectance, optical coherence tomography

## Abstract

**Significance:**

Atrial fibrillation is treated with thermal ablation to isolate ectopic signals. Although this is the current standard of care, recurrence occurs in up to 40% of cases. Clinicians have no reliable way to predict treatment durability intraoperatively. Adding the capability of direct optical measurement of the tissue to an ablation catheter could help better guide the treatment.

**Aim:**

A radiofrequency ablation catheter was developed with polarization-sensitive optical coherence tomography (PSOCT) and near-infrared spectroscopy (NIRS) to demonstrate, for the first time, simultaneous monitoring of thermal lesion formation.

**Approach:**

We fabricated the multimodal PSOCT-NIRS ablation catheter and validated optical metrics using known targets of tissue-like phantom, deoxygenated blood, and atrial tissue. We then demonstrated recording PSOCT and NIRS data during *ex vivo* ablation of swine atria.

**Results:**

PSOCT-NIRS metrics showed expected values in known targets. Measurements during ablation also exhibited previously reported patterns—OCT scattering increases, PSOCT birefringence decreases, and NIRS lesion optical index increases. Furthermore, simultaneous measurement revealed varying rates of change and magnitudes of response to different powers of thermal ablation.

**Conclusions:**

PSOCT-NIRS can measure tissue response to thermal energy delivery, and the optical metrics are complementary. By collecting more information during thermal energy delivery, PSOCT-NIRS metrics could contribute to understanding treatment durability in future investigations.

## 1 Introduction

Atrial fibrillation (AF) is the most prevalent arrhythmia, affecting nearly 60 million people worldwide.^1^^–^^3^ AF is an irregular beating of the heart’s upper chambers caused by the abnormal generation of electrical signals, which is readily identified using an EKG. However, patients can experience varied responses to AF, from asymptomatic to substantially reduced quality of life.^4^^,^^5^ Whether symptomatic or not, the AF dramatically increases the risk of ischemic stroke,^6^^–^^8^ and AF-related deaths have surpassed 300,000 annually.^3^ The global impact of AF doubled from 1990 to 2019, and this trend is expected to continue over the next three decades.^1^^,^^9^ Although AF can be deadly, various management modalities exist, both pharmacologic and procedural, for either the goal of rhythm or ventricular-rate management.^10^

Catheter ablation of AF is the current standard-of-care.^11^^,^^12^ The most frequently used ablation therapy treats AF with radiofrequency (RF) current, causing therapeutic thermal lesions. RF catheters are typically equipped to measure contact force, impedance, and temperature and are irrigated to prevent excess heat or steam formation.^13^ The operating clinician chooses the power and the duration of RF energy to achieve the intended thermal damage to the myocardium. This ablation prevents electrical activity and conduction, with the goal to electrically isolate or eliminate arrhythmogenic regions of the atrium to block the initiation of ectopic beats. The typical lesion pattern, pulmonary vein isolation, surrounds the pulmonary veins, the most common ectopy source for AF.

RF ablation of AF is an effective treatment, but recurrence of the disease after the procedure remains high. Recurrence occurs in 30% to 40% of patients just 1 year after treatment.^12^^,^^14^ Recurrent AF can lead to myocardial remodeling that worsens the condition, and the result is that catheter ablation is not curative for many patients with AF.^15^^–^^22^ Intraoperative feedback is limited and lacks monitoring of lesion transmurality (ablation completeness through the atrial wall thickness). Limited feedback may lead to lesion-to-lesion errors, with incomplete lesions failing to isolate the intended myocardium and excessive energy potentially damaging surrounding tissue. Tools to predict the durability of the treatment during the operation may reduce this recurrence rate. Gaps in lesion lines and nontransmurality contribute to these nondurable outcomes, and the current technology does not detect these critical failures during the procedure. No current method provides a complete picture of long-term treatment success.^23^

To provide operator feedback, some ablation devices calculate new indices as a proxy for ablation completeness or diameter.^24^^–^^29^ These indices incorporate combinations of power and duration of energy delivery, impedance, and contact force. They are limited because they only inform the clinician about the energy delivery, not the tissue’s response. Current research uses novel sensing at the ablation site to try to enhance feedback.^30^^–^^36^ Our previous work has shown that direct tissue measurements using optical techniques can visualize and predict lesion parameters. Catheter-based polarization-sensitive optical coherence tomography (PSOCT) can immediately indicate tissue response to RF energy delivery as birefringence of myocardial tissue decreases and scattering increases during ablation, as previously demonstrated both *in vivo* and *ex vivo*.^37^^–^^43^ Near-infrared spectroscopy (NIRS) measures spectral changes of light diffusely backscattered from tissue as RF energy is delivered to predict lesion depth and diameter.^44^^–^^48^ Both techniques can work in tandem, providing complementary feedback. High-resolution imaging gained from PSOCT identifies clinically relevant sub-millimeter gaps in lesion lines, as well as showing morphological detail of endocardium, adipose, and very thin tissue. NIRS can predict lesion depth in all clinically relevant regions of the atria, beyond the depth range of OCT, but the technique does not provide an image. Together, PSOCT and NIRS can provide a robust view of the left atrial tissue during treatment.

Here, we demonstrate a novel catheter that simultaneously ablates and measures PSOCT and NIRS. This mitigates the limitations of each technology and also provides opportunities for new multimodal measurement synergies. We describe the catheter design and present measurements of phantoms and tissue targets to validate the functionality of the multimodal catheter. Then, we demonstrate optical data collection during *ex vivo* ablations to further demonstrate the optical measurements’ utility for understanding ablation for atrial arrhythmias.

## 2 Methods

### 2.1 System Description

**Fig. 1:**
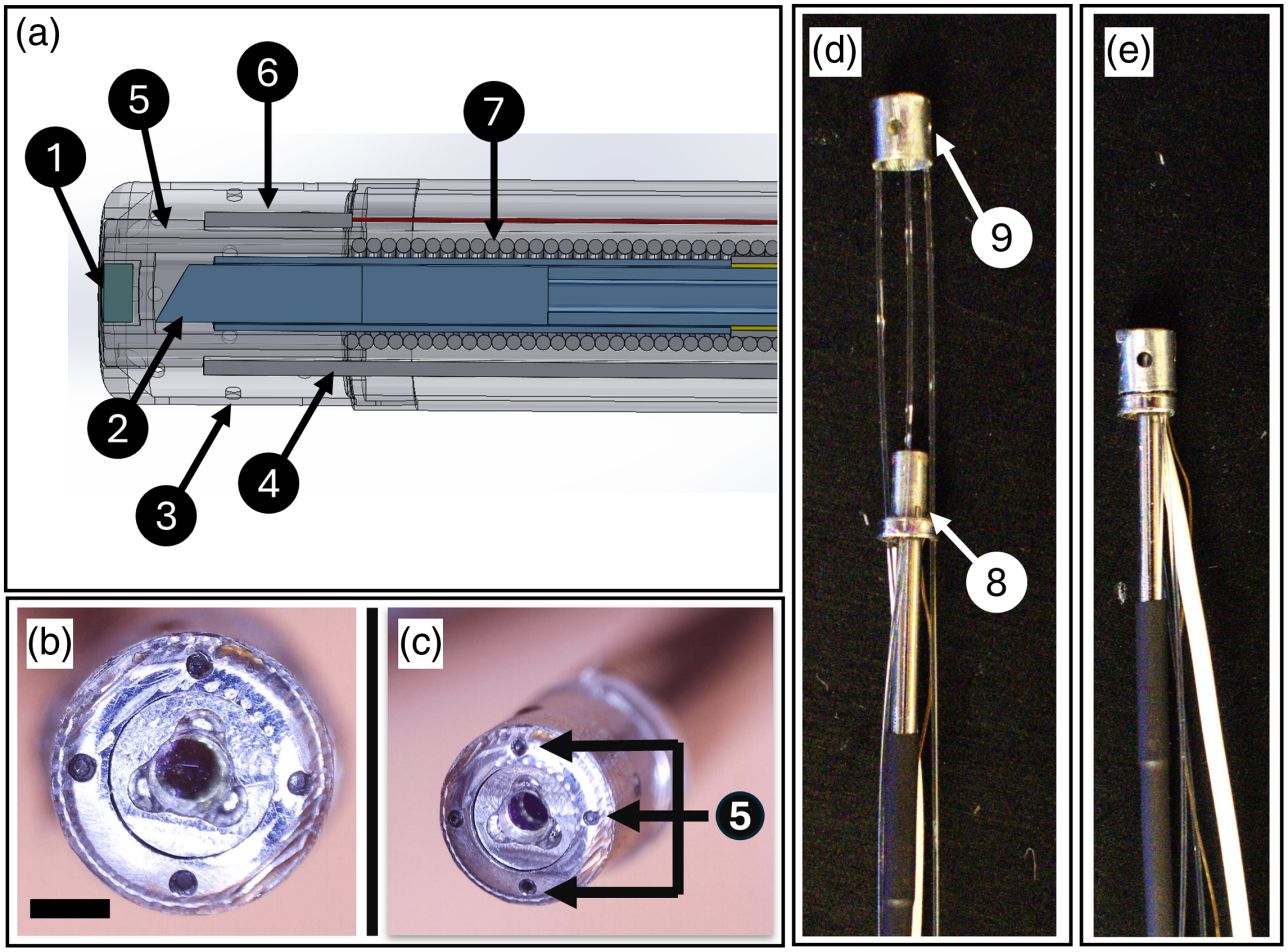
In the CAD cross-section (a), the components of the probe are labeled: (1) glass window, (2) GRIN lens of the PSOCT probe, (3) irrigation ports, (4) radiofrequency electrode, (5) NIRS fiber, (6) thermocouple, and (7) torque coil. Band electrodes are included in the fabrication but are not shown. Panel (b) provides a head-on view of the glued window glass and the circumferential NIRS fibers, which are also labeled as (5) in panel (c). The scale bar measures 1 mm. In panel (d), the entire tip is divided into the insert electrode (8) and the NIRS shell (9). This panel reveals the exposed fibers. In panel (e), the two components are securely fixed together.

In addition to the optics, the catheter features standard interfaces for use with clinical ablation and mapping instrumentation. These are visualized in a CAD drawing in Fig. 1(a) and include a thermocouple (6), irrigation ports (3), and a radiofrequency electrode (4). We have designed a custom connection and software for the multimodal catheter to interface with a commercial ablation unit (Boston Scientific, Maestro 3000). This allows us to record power, impedance, and temperature throughout the ablation time. A lumen and weep holes enable irrigant to cool the catheter tip. We have also fabricated the catheter sheath with band electrodes and verified that they interface with a cardiac mapping system (Biosense-Webster, Carto, Irvine, California, United States). This enables the clinician access to current *in vivo* positioning capabilities with this catheter.

We designed the multimodal catheter with a central PSOCT probe and circumferential NIRS fibers built into the ablation electrode to simultaneously record optical metrics at the same location [Fig. 1(a), annotations 2 and 5]. We organized the tip into two primary components: the insert electrode [Fig. 1(d), 8] and the NIRS shell [Fig. 1(d), 9]. The NIRS shell provides a glue joint for all NIRS fibers (Thorlabs, FG200LEA, Newton, New Jersey, United States) and open-irrigation holes to pump saline to keep the tip cool. The separated NIRS shell lets us polish the fibers without fouling other optical components or joints in the insert electrode. The insert electrode has machined slots for glue joints that hold the thermocouple, radiofrequency electrode, and irrigation port in place. Furthermore, guides for the NIRS fibers serve as strain relief to prevent the fibers from breaking. The insert electrode contains features to center the PSOCT probe and maintain its distance from the back face of a glass window in the ablation electrode tip. Drilled and reamed steel tubes and machined ledges in the insert electrode provide a bearing surface during rotation. The window is fixed with optical adhesive (Norland Optical Adhesive, 89H, Cranbury, New Jersey, United States) with glue squeeze-out channels separated 120 deg around the window. The NIRS shell and insert electrode are then fixed together, and the only rotating component is the PSOCT probe within the catheter tip.

The PSOCT probe is designed to focus to a 16  μm spot 0.5 mm beyond the forward face of the window.^49^ A torque coil (Asahi) transfers the rotation from an FC/APC connection to an optical subassembly. The optical subassembly is made from a steel tube that centers a coreless spacer for a concentric glue joint (Norland Optical Adhesive, 86H) to a GRIN lens. The GRIN lens and spacer are fabricated with an 8 deg angle to reduce back reflection at the interface. Once the GRIN lens is secured to the spacer, the distal face of the GRIN lens is polished to achieve the designed focal length. The GRIN lens is polished at a 20.5 deg angle using a custom fixture to direct the beam 11 deg off-axis. The polish angle can be adjusted to achieve different diameters of the conical scan. For this probe, the diameter of the scan at the focus is 1 mm. Polishing angle and focal distance are reproduceable using fixtures to hold the optical subassembly, and success is measurable with a beam profiler.

Our PSOCT system consists of a 50 kHz swept source laser (Axsun) with a frequency-doubled k-clock sampling trigger to enable sufficient range for depth-multiplexed orthogonal polarization illuminations via a polarization delay unit. We use a fiber-based polarization-diverse detection to capture all Jones matrix values simultaneously. The central wavelength of the laser is 1308 nm with a bandwidth of 146 nm, and the predicted axial resolution is around 5  μm in air. We are using a DAQ (AlazaarTech, ATS9371-4G, Pointe-Claire, Canada) to record signals from two balanced differential photodetectors (Thorlabs, PDB470C-AC). The system description has been reported previously.^37^

Local birefringence and intensity values were calculated after data collection using previously documented methods.^50^ In short, the interferograms are processed to construct OCT A-lines. Then, the Jones components are aligned to subpixel resolution by applying a phase ramp to the depth-multiplexed image. The Jones matrix is used to calculate the local birefringence. The local birefringence is assumed to be a linear retarder and is a measure of the magnitude of the retardance at each pixel depth.

The NIRS illumination source is a Tungsten-Halogen lamp (Ocean Optics, HL-2000-HP, Orlando, Florida, United States). The spectrometer has a customized slit size of 200  μm (Hamamatsu, C9405CCMOD, Hamamatsu, Japan) to increase measurement throughput. The NIRS fibers in this catheter design have a maximum source detection separation of 3 mm. However, for this demonstration, we use a 2 mm source-to-detector separation. A previously published study used a catheter with a similar (2.3 mm) source-to-detector separation and developed a reliable calculated index to predict lesion formation in the left atria.^51^ This lesion optical index (LOI) is used in the validation experiment reported here and consists of the ratio of relative reflectance of 964 and 616 nm. Relative reflectance is calculated using standard calibration: removing the dark spectra, removing the instrument response, and normalization with known phantom optical properties.^44^ Spectra are collected with 10 ms integration time.

### 2.2 PSOCT & NIRS Validation

We characterized the PSOCT and NIRS functionality of the catheter by performing validation tests on known targets. To show that the PSOCT works as expected, we used the catheter to image a previously described tissue-like birefringent phantom with a gradient of birefringence values along its length.^52^ The phantom has a tissue-like backscattering signal, and the range of birefringence values is comparable to muscle and collagen.^52^^–^^54^ Specifically, the phantom helped validate ablation monitoring capability because it included birefringence values within the range measured in the atrial tissue before and after ablation. Briefly, we annealed a strip of polycarbonate and prepared a tapered section. The phantom’s original edge width measured 20 mm, tapering to 6 mm, and its original full length was 70 mm. We then stretched the polycarbonate in a convection oven to enforce even strain along the length. As shown previously, we expect the birefringence to scale inversely with the width of the stretched phantom.^52^ Using the catheter, we measured birefringence and backscatter PSOCT images at several taper widths, corresponding to several different birefringence values.

To validate the NIRS functionality, we measured relative reflectance spectra of heparinized deoxygenated blood and swine tissue. The reflectance spectrum of blood is a good validation target for NIRS as it shows an important absorption peak corresponding to deoxyhemoglobin at around 750 to 760 nm. The blood and tissue measurements were made with PSOCT and NIRS to show that the simultaneous measurements both confirm the transition from blood to tissue contact.^51^

### 2.3 *Ex Vivo* Ablation

To demonstrate the catheter’s capability to ablate the atrial wall and simultaneously monitor the ablation, we acquired fresh swine atria from a local abattoir (3D Meats, Dalton, Ohio, United States). We then ablated the tissue while simultaneously collecting PSOCT, NIRS, and ablation unit data in real time. The ablation unit provided power, temperature, and impedance data via an RS232 connection. Temperature and power remained constant throughout the duration of energy delivery. Each ablation lasted 60 s, with the powers set at 5, 10, 15, and 20 W. Recording of optical metrics started 5 s before energy delivery began and continued until 10 s after energy delivery stopped. An experimental rig maintained the catheter at a consistent force and right-angle apposition to the atrial tissue. The tissue was kept in a bath of phosphate-buffered saline (PBS) at 37°C. A pump circulated the PBS across the tissue, modeling *in vivo* blood flow. Room temperature PBS served as the irrigant. Custom software was used to synchronize data collection and ablation timing. Irrigant was forced through the catheter using a syringe pump to maintain a flow rate of 15 mL/min. Intensity and birefringence values are reported as the mean and median, respectively, from a manually segmented region in each frame, extending from beneath the endocardium to the deepest imaged myocardial depth. PSOCT images were recorded at 10 frames per second, and NIRS spectra were recorded at 10 Hz. Subsequently, we stain the tissue with 2,3,5 triphenyl-tetrazolium chloride (TTC)—a consistent gold standard in the field used to identify RF-treated regions of tissue.^55^^–^^57^

## 3 Results

**Fig. 2:**
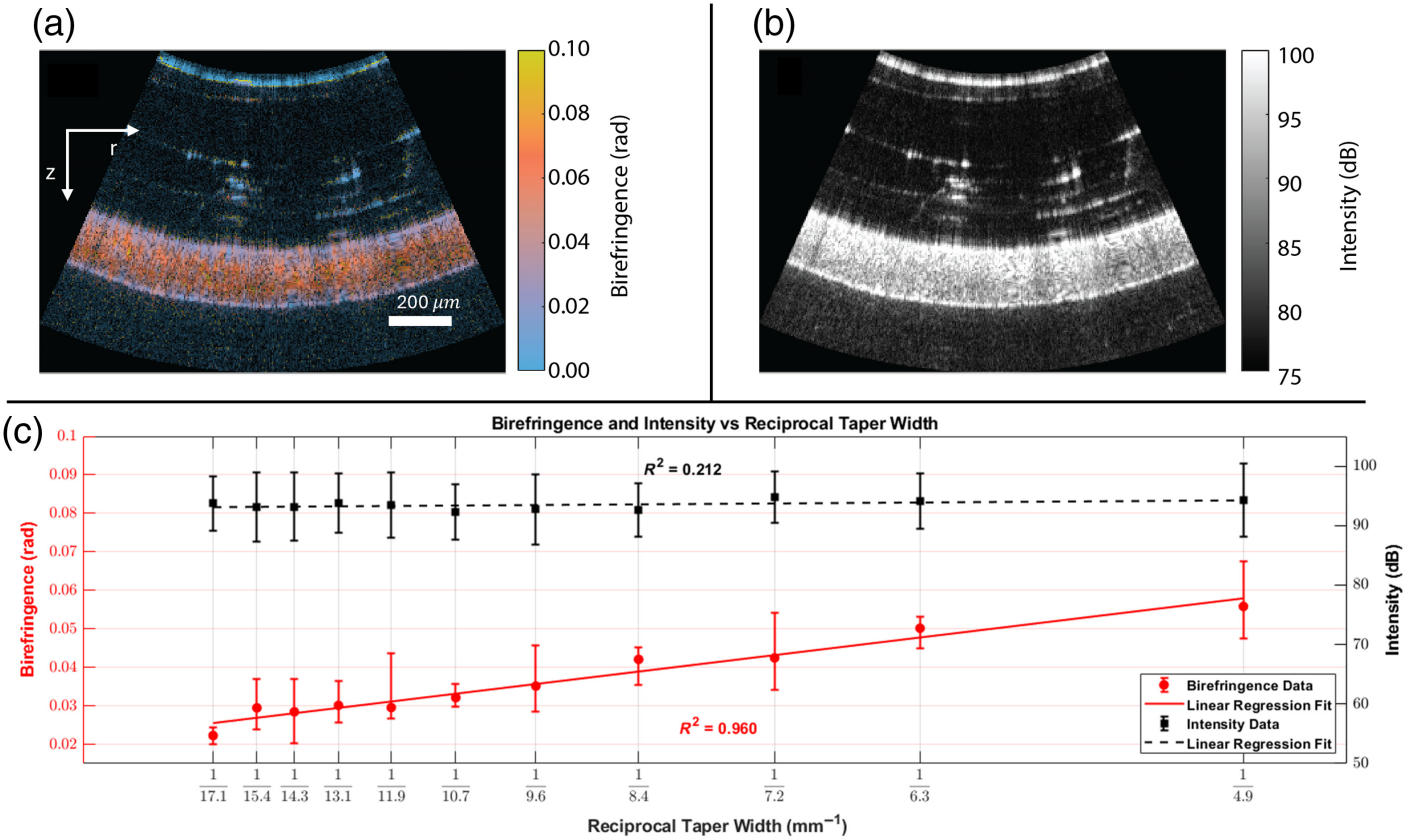
Panels (a) and (b) show example birefringence and intensity images, respectively, of a polycarbonate phantom. Birefringence increases along the length of the phantom as taper width decreases, whereas scattering intensity remains unchanged. A strong linear relationship exists between birefringence and the inverse of taper width [red line in panel (c)], with an $R^{2}$ value of 0.960. Intensity has poor correlation with taper width [black line in panel (c)] with an $R^{2}$ value of 0.212.

Both the PSOCT and NIRS functioned as expected in this multimodal catheter. To validate the PSOCT, we imaged along the length of a phantom with a known relationship between birefringence and location on the phantom. Higher birefringence values are measured in locations that experience higher stress on the polycarbonate phantom. In Fig. 2, panels (a) and (b) show example B-scans at a single location. The images of birefringence and intensity represent a conical scan, with the axes labeled as z and r. In Fig. 2(c), we show that birefringence exhibited the expected linear correlation with inverse taper width ($R^{2}=0.960$). By contrast, the location on the phantom accounted for only ∼21.2% of the variation in the conventional intensity images ($R^{2}=0.212$), indicating that the PSOCT is sensitive to changes in birefringence that are not accompanied by changes in backscatter intensity. Birefringence and intensity are plotted as the median and mean values, respectively, in the manually segmented region of the image. Error bars show the inner and outer quartiles for birefringence and the standard deviation in the mean for intensity.

**Fig. 3:**
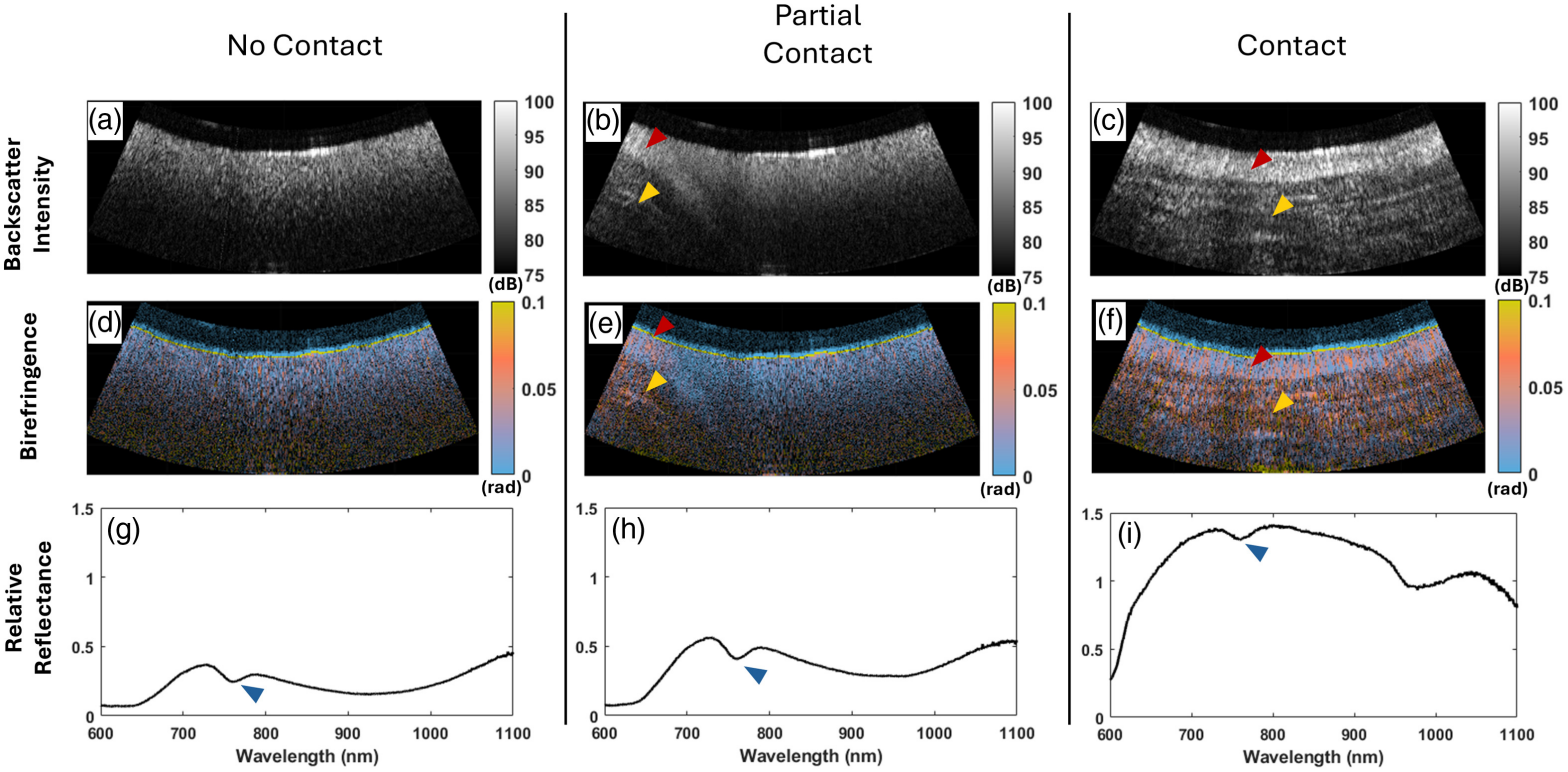
Simultaneous measurements indicate that PSOCT and NIRS can detect tissue contact when the left atrium is submerged in blood. The conventional OCT (a)–(c) and birefringence (d)–(f) images display the blood pool and the tissue as the catheter approaches tissue contact. The blood appears homogenous and nonbirefringent (a), (d). The lower birefringent endocardium (red arrow) and the higher birefringent myocardium (yellow arrow) are partially in frame as the catheter makes partial contact with the atrial wall (b), (e). During complete wall contact, the images show a heterogeneous structure in the myocardium (yellow arrow). The NIRS relative reflectance (g)–(i) is of lower magnitude when the catheter is floating in the blood pool compared with tissue contact. In addition, the absorbance peak from blood constitutes a larger proportion of the signal, as annotated with a blue arrow in panels (g)–(h).

To validate the NIRS measurements, we show that the measured spectra differentiate blood from tissue in Fig. 3. The simultaneous measurements of PSOCT and NIRS are in agreement, as contact with the tissue is readily visible with both techniques. It is clear to see the tissue approach the catheter in the OCT images [Fig. 3(a)–3(c)]. The tissue has a heterogeneous structure compared with the image of the blood pool, and in the example in Fig. 3, the endocardium is seen as the highly scattering layer that is lowly birefringent [red arrow in Fig. 3(b), 3(c), 3(e), and 3(f)]. By contrast, the myocardium has a higher birefringence and a heterogeneous structure [yellow arrow in panels (b), (c), (e), and (f)]. The NIRS measurements of relative reflectance further verify tissue contact. In panels (g), (h), and (i), deoxygenated hemoglobin exhibits a known absorption peak between 750 and 760 nm.^58^ We can see this as a dip in the relative reflectance of blood (blue arrows). When only the blood pool is present, the contributing magnitude of the blood absorption is much larger than when the catheter is in contact with the tissue. Furthermore, the relative reflectance increases as the probe samples atrial tissue instead of blood, which agrees with the previously reported spectral shape.^48^

**Fig. 4:**
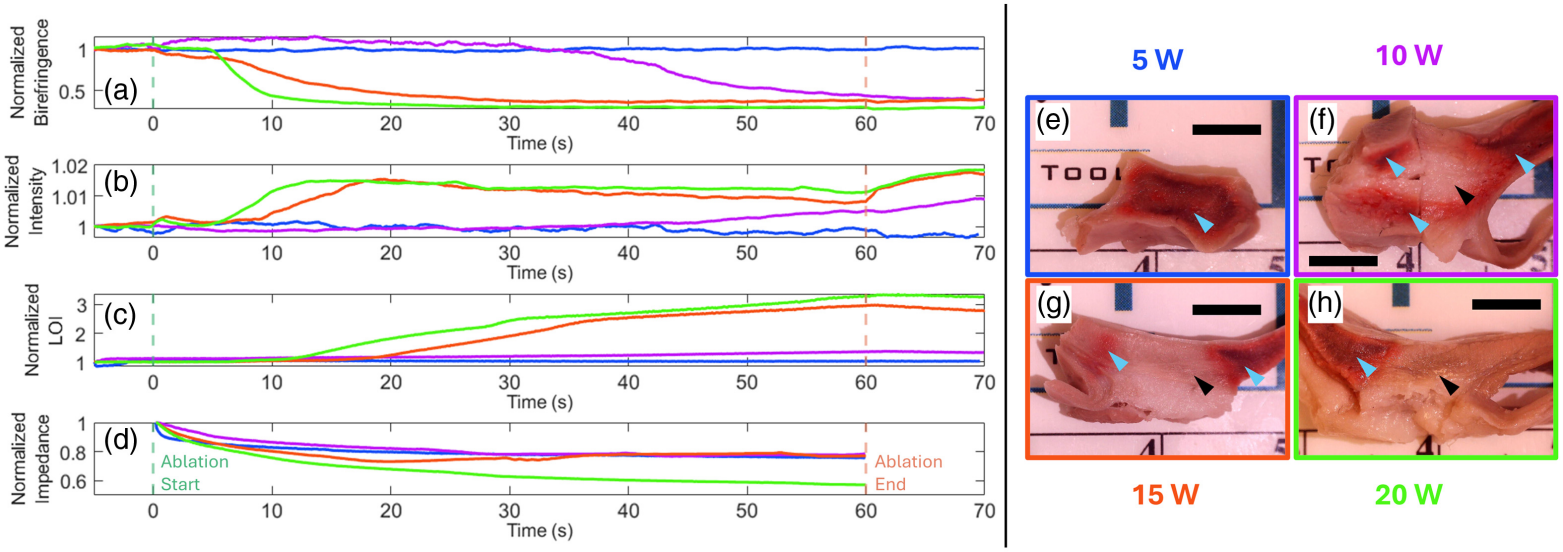
Four lesions were ablated in the swine atrium *ex vivo*. Each lesion was treated for 60 s, with different powers (5, 10, 15, and 20 W). The patterns measured by optical metrics (a)–(c) and the standard impedance metric (d) follow trends related to ablation power. Birefringence (a) decreases as ordered proteins denature with energy delivery, and the 20 W lesion also decreases the most and the fastest. Intensity increases as the scattering properties of the tissue change (b), and the 20 W lesion increases the most and the fastest. The NIRS LOI (c) increases as spectral changes indicate lesion formation. Impedance (d) decreases more as higher energy is deposited. The TTC staining reveals the treated region in pale white (black arrows), whereas untreated regions appear a deeper red (light blue arrows). All lesions were ablated in the same area of the left atrium in the same swine heart, and the scale bar is 5 mm. However, the 5 W lesion did not leave a visible treated lesion in the atrial wall. The optically derived metrics (a)–(c) show minimal change during the 5 W energy delivery.

The multimodal catheter can simultaneously collect optical metrics derived from PSOCT and NIRS, as well as standard ablation metrics, and we demonstrate measurements made during ablation at four different RF powers in Fig. 4. This *ex vivo* demonstration illustrates the potential utility of the multimodal catheter. In Fig. 4(a)–4(c), we present PSOCT, OCT, and NIRS metrics traced over the time course of energy delivery and lesion formation. Panel (d) shows the measured impedance as energy is delivered, which is standard in clinically available catheters. All metrics are normalized to their initial values. PSOCT birefringence decreases as thermal energy is deposited. OCT scattering intensity increases. The measured NIRS LOI increases during energy delivery as well. Impedance decreases. The TTC staining of each lesion, shown in panels (e)–(h), highlights the affected area of each treatment as a pale region within the atrial wall thickness, also shown by a black arrow. The dark red indicates viable tissue and corresponds to areas not treated with thermal energy, also annotated with a light blue arrow. By observing the pale regions in images, we see that the 15 and 20 W ablations are transmural. The 10 W ablation is nontransmural. There is no evidence of treatment in the cross-section of the 5 W lesion.

## 4 Discussion

This multimodal catheter will enable future *in vivo* large animal studies to further investigate the synergy of PSOCT and NIRS in guiding the treatment of atrial arrhythmias. Although monitoring RF ablation of the atrial wall using OCT, PSOCT, and NIRS has been previously demonstrated, this catheter design enables simultaneous multimodal monitoring of ablation treatment for the first time. Here, we have shown concurrent PSOCT and NIRS measurements of both catheter-tissue contact and of ablation. This catheter will be used in future *in vivo* studies to further assess the capabilities of PSOCT-NIRS metrics to assist in the ablation treatment of arrhythmias.

The catheter was constructed with careful CNC machining and manual optical assembly. The resulting device is well suited for research and development, allowing for customizability in a workflow with changing requirements. The catheter presented here is capable of *in vivo* research studies and points the way toward design for further miniaturization and scaled-up manufacturing.

The validation of birefringence measurements with the tissue-like tapered polycarbonate phantom shows that the current catheter can measure a range of different values (Fig. 2). For the application of ablation monitoring, a decrease in birefringence indicates thermal damage to the tissue. PSOCT can also measure the optic axis orientation of birefringent materials, but making this measurement using a catheter requires additional signal reconstruction methods and is the subject of future work. Scattering is also essential to measure in conjunction with birefringence. We did not expect any change in the OCT intensity along the length of the phantom. The results lend confidence that there are no artifacts in birefringence measurements caused by variations in intensity.

We measured atrial tissue submerged in blood to validate the NIRS functionality of the catheter while simultaneously visualizing contact with PSOCT imaging. The expected absorbance peak of blood between 750 and 760 nm is clearly visible in the relative reflectance spectra [blue arrow in Figs. 3(g), 3(h), and 3(i)]. Furthermore, the spectral shape of the atrial tissue [Fig. 3(i)] matches previously reported measurement values.^48^ Effective ablation requires direct tip to tissue contact. Contact sensing is an important capability for the ablation workflow. As shown in Fig. 3, PSOCT-NIRS readily assesses differences in partial and complete contact. The measurements have been tested individually and have shown that PSOCT^42^ and NIRS^46^ can reliably measure tip apposition to the atrial wall.

Finally, ablation of the LA wall at four different power levels shows that optical metrics—OCT scattering, PSOCT birefringence, and NIRS LOI—follow trends in tissue response to energy delivery. For the 10, 15, and 20 W ablations, OCT scattering and NIRS LOI increase as PSOCT birefringence decreases. Birefringence values decrease by over 50% during energy delivery; however, the rate of change corresponds to the power of the ablation. The 20 W ablation reaches steady state the fastest, followed by the 15 W and then the 10 W ablation. OCT scattering increases during the energy delivery, but the magnitude of change is much smaller (1% to 2%). The rates of change, though, follow the same correspondence to energy delivery as birefringence, with the 20 W ablation leading to the fastest change.

The NIRS LOI also shows contrast to energy delivery equivalent to contrast previously observed using stand-alone NIRS probes with a similar source-to-detector distance. For the NIRS LOI measurement of the 10, 15, and 20 W ablations, similar relative rates of change are reported. Again, the 20 W ablation shows the fastest change. It is notable that PSOCT birefringence and OCT scattering reach steady state at different times from the NIRS LOI at the same energy delivery. In the case of the 20 W ablation, scattering and birefringence reach a steady state close to 10 s after energy delivery is initiated. NIRS LOI lags, showing a slower rate of change at 30 s, but it never reaches a steady state.

In the case of the 10 W ablation, it is interesting to note that the birefringence reaches the same magnitude of change as the 15 and 20 W ablations. The NIRS LOI, on the other hand, has noticeably different endpoints for each ablation. Specifically, for the 10 W ablation, there is a 30% increase in the NIRS LOI, whereas for the 15 and 20 W ablation, the ending value is three times the beginning value. This could be due to a partial volume effect. Note that the TTC stain for the 10W lesion [Fig. 4(f)] indicates nontransmurality. This NIRS LOI pattern, along with the ground truth evidence from TTC staining, could indicate that the NIRS probe was sampling both treated and untreated tissues, leading to the relatively modest change in the NIRS LOI at 10 W power delivery. TTC staining can assess lesion completeness; however, the overarching goal is to predict durability. Future survival studies aim to assess the lesion durability of PSOCT-NIRS-informed treatment. Durable lesion sets will decrease AF recurrence.

Another important advantage of PSOCT-NIRS is clear when inspecting the 5 W ablation in Fig. 4. First, the TTC staining in panel E shows no clear evidence of treatment. However, the impedance drop [panel (d)], the current clinical metric used to terminate energy delivery, shows that the pattern for the 5 W ablation is nearly equivalent to the 10 and 15 W ablations. It is important to make a distinction between measuring current delivery and measuring tissue damage. In the 5 W ablation, impedance drop measures current delivery and likely tissue heating; however, PSOCT-NIRS is directly measuring the tissue. OCT scattering, PSOCT birefringence, and NIRS LOI show no change over the course of energy delivery with the 5 W ablation. In this demonstration, PSOCT-NIRS would accurately predict that the 5 W ablation would not thermally damage the tissue, but impedance drop alone would not. In all lesion power examples, OCT intensity, PSOCT birefringence, and NIRS LOI follow expected trends with RF power but exhibit different magnitudes and time courses of change. This is consistent with the idea that they are complementary measurements, sensitive to multiple tissue optical properties (scattering, birefringence, absorption). This may enable building a robust predictor of lesion completeness and durability by investigating interactions between the measurements. Future studies will aim to use a larger sample size of lesions to quantitatively demonstrate the combined prediction of PSOCT and NIRS metrics in assessing lesion transmurality.

## 5 Conclusion

We have developed a multimodal PSOCT-NIRS catheter, confirmed critical measurements, and tested its utility in an *ex vivo* ablation experiment. This will facilitate future experiments that will address questions about the synergy of the PSOCT images and NIRS spectra. In addition, this catheter is expected to facilitate the translation of PSOCT and NIRS cardiac ablation therapy by enabling large-animal *in vivo* ablation studies.

## Data Availability

All data in support of the findings of this paper are available within the article.
